# Modeling the Distribution and Environmental Preferences of the Ladakh Urial in the Arid Himalayas

**DOI:** 10.1002/ece3.70423

**Published:** 2024-10-09

**Authors:** Jeremy Roy Lambe, Mohd Raza, Tsewang Namgail

**Affiliations:** ^1^ Snow Leopard Conservancy India Trust Leh Ladakh India

**Keywords:** arid, cold, desert, India, Ladakh, species distribution, steppe, urial

## Abstract

Mountains play a crucial role in shaping the climate of an area and subsequently, the environments and species that are suited to those particular conditions. Understanding the relationships between environmental conditions and their influence on the occurrence of a species is necessary to make informed decisions when conserving relevant habitats. This study evaluated and compared the potential for different interactions between climatic variables to describe the environmental preferences and the range of the Ladakh urial, a narrowly distributed sub‐species of *Ovis vignei*, in the arid Himalayas. Species distribution models were created from spatially thinned presence‐only occurrence data of 50 locations using a Maximum Entropy (Maxent) model. For this, we subdivided the modeled distribution into environmental categories with varying degrees of suitability. We identified a high cold precipitation to annual precipitation ratio typical of cold desert climates well suited to describe the highest quality environments for urial. The inverse, low warm precipitation to annual precipitation ratio was found to better describe the urial's range at the lower end of potential habitat. In this comparison, we observed that with the exclusion of unsuitable cold steppe habitat with a higher warm precipitation to annual precipitation ratio, we were able to refine the extent of this species range but at a trade‐off to the accurate description of high‐quality environments. This study demonstrated the strong potential of cold arid climatic conditions to describe the distribution of urial in India, which have implications for identifying important areas for the conservation and management of the species in the high altitude rangelands of Ladakh.

## Graphical abstract

This study contributes to the understanding of the habitat selected by a vulnerable sub‐species with a highly restricted range influenced by local climatic conditions.

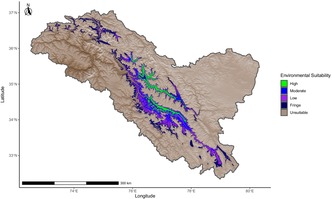

## 1 Introduction

As human activities increasingly alter and encroach on wildlife habitat worldwide, it is more important than ever that these habitats are well understood so that informed conservation strategies can be developed to maintain wild populations and minimize human‐wildlife conflict (Pardini, Nichols, and Püttker 2017). This is especially true in mountain environments where habitats are already fragmented by topography and dramatic elevation gradients leaving populations more susceptible to isolation (Steinbauer et al. 2016). The Ladakh urial *Ovis vignei vignei*, locally known as Shapo, is a species that has a restricted distribution within a limited number of valleys in India, most notably in the Indus, Shayok, and Nubra valleys (Fox, Nurbu, and Chundawat 1991; Namgail et al. 2010; Michel and Ghoddousi 2020; Khara et al. 2021).

In the valleys where the Ladakh urial occurs, it can often be seen utilizing flat, open areas, but also spending time in the rugged terrain around cliffs (Fox, Nurbu, and Chundawat 1991; Raghavan, Bhatnagar, and Qureshi 2003; Namgail et al. 2010; Michel and Ghoddousi 2020; Khara et al. 2021). Here they forage on woody shrubs, forbs, and grasses where they may be in competition with other ungulates, both wild and domestic (Fox, Nurbu, and Chundawat 1991; Raghavan, Bhatnagar, and Qureshi 2003; Namgail et al. 2010; Michel and Ghoddousi 2020; Khara et al. 2021). Due to their utilization of the flats along valley bottoms, urial in India also face considerable threats from development, which also favors these flat areas. Through activities such as rural and municipal expansions, infrastructure projects, and as a result of militarization in the region, these valleys have seen dramatic changes (Fox, Nurbu, and Chundawat 1991; Raghavan, Bhatnagar, and Qureshi 2003; Namgail et al. 2010; Khara et al. 2021). Because of the threats facing the small, isolated populations of urial that are seeing a declining trend worldwide, this species is listed as vulnerable under the IUCN Red List of Threatened Species (Michel and Ghoddousi 2020). Although human activities have likely influenced this species' distribution, it is valuable to establish a baseline of potentially suitable habitats so that the natural disposition of these animals can provide context when addressing their contemporary occurrence among altered habitats.

These striking sheep with full‐grown males boasting a dark neck ruff and distinct horns that originate high and forward on the head play an important role both culturally and ecologically. In Ladakh, urial have historically been hunted for food and used as sources of materials, ornamentation, and medicines (Bhatia et al. 2021; Hassan et al. 2022). In the broader ecosystem, urial serve as the primary consumers of Ladakh's arid, and sparsely vegetated environments (Khara et al. 2021). Because of its propensity to use relatively rugged as well as flatter areas, urial are preyed on by predators such as Himalayan wolves *Canis lupus chanco* occurring in flat areas and snow leopards *Panthera uncia* inhabiting rugged areas (Namgail et al. 2010; Habib, Shrotriya, and Jhala 2013; Watts, McCarthy, and Namgail 2019; Balajeid Lyngdoh, Habib, and Shrotriya 2020). Therefore, we look to broader trends in the urial's environment to help describe their narrow distribution.

As grazers, urial rely more on graminoids than other sympatric species such as the blue sheep *Pseudois nayaur* (Namgail et al. 2010). The primary productivity of a particular area and the species of plants found there are strongly influenced by the climate of that area (Churkina and Running 1998). Just as plants have specific tolerances to climatic conditions, some herbivores may also have similar range limiting tolerances for the abundance, type, and quality of vegetation available to them. Furthermore, if a species of plant grows under certain climatic conditions, we should expect a similar pattern to be followed by the herbivores that consume those plants. Many species of plants in Ladakh may grow optimally in places where the precipitation falls mostly in the form of snow and less in the form of rain, which can wash away the substrate from steep slopes. Snow on the other hand can seep gradually into the ground, maintaining a constant supply of moisture (Dvorský et al. 2011; Kumar, Singh, and Sharma 2005). We hope to model the urial's distribution from this relationship between consumer and climate, mediated by the producer.

Ladakh's climate is largely influenced by the high mountain ranges that lie within its territory. The arid valleys in the rain shadows of these massive mountain ranges are primarily where urial are found in Ladakh. This general observation leads us to our hypothesis that Ladakh urial are primarily adapted to an arid environment, specifically a cold desert climate, and that their distribution can be described by the meteorological conditions that lead to this arid environment, namely precipitation and temperature. In this study, we examined the environmental conditions that are favorable to urial by modeling the presence of this species in relation to climatic variables to gain insights into the niche that urial inhabit and the extent to which that niche can describe their restricted distribution.

## 2 Materials and Methods

### 2.1 Data Collection

Presence‐only data were collected through visual surveys conducted in Sham, Kargil, and Nubra by recording the locations of individuals or groups of urial. These surveys were conducted by multiple observers (typically 2 or 3) while walking along trekking trails or driving and involved systematically scanning valleys with the aid of binoculars and spotting scopes. On the days that surveys took place at least two daylight hours were spent actively surveying, and surveys were not done with a set frequency. Urial observed incidentally (while not actively surveying) were also recorded. As surveys were performed while traversing established roads and trails a potential detection bias was introduced by sampling more frequently from accessible areas. Location coordinates were collected by projecting and recording points in GPS units based on compass bearings and landscape features from topographic maps.

### 2.2 Climatic Variables

We examined representative climatic variables used in the definition of the cold desert climate (BWk), thought to be the primary habitat of Ladakh urial, as well as the other predominant arid subgroup cold semi‐arid (steppe, BSk) climate in Ladakh under the Köppen‐Geiger climate classification system (Peel, Finlayson, and McMahon 2007). In the Köppen‐Geiger classification desert (BW) and steppe (BS) climates are defined by a relationship between annual precipitation, 6‐month seasonally dependant precipitation, and temperature; cold (k) climates are defined as having mean annual temperatures less than 18°C, as outlined in Table 1 of Peel, Finlayson, and McMahon (2007). The climatic variables tested reflected those needed to identify BWk and BSk climates and included mean annual temperature (MAT), mean annual precipitation (MAP), precipitation of the coldest quarter (*P*_cold_), and precipitation of the warmest quarter (*P*_warm_) obtained as rasters with a resolution of ~1 km^2^ on a WGS 84 projection from www.worldclim.org (Fick and Hijmans 2017). Cold desert climate was best represented by the combination of MAT, MAP, and *P*_cold_ variables, and cold steppe climate was best represented by MAT, MAP, and *P*_warm_ variables. The *P*_cold_ and *P*_warm_ variables were adapted from the original Köppen‐Geiger system of 6‐month summer (AMJJAS) and winter (ONDJFM) precipitation periods to better isolate the phases (liquid and solid) of precipitation. The 6 month summer and winter precipitation periods were used to report the Köppen‐Geiger climate category at presence locations, but only the quarterly summer and winter precipitation periods were used in modeling.

**TABLE 1:** Urial distribution models with accuracy evaluation using the Area Under Curve (AUC) of randomly assigned Train and Test data subsets, and model ranking using Akaike Information Criterion corrected for sample size (AIC_c_) and the difference from the top model (Δ AIC_c_).

Model	AUC Train	AUC Test	AIC_c_	Δ AIC_c_
MAT + MAP + *P*_cold_ + *P*_warm_	0.997	0.995	675.045	0.000
MAT + MAP + *P*_warm_	0.997	0.997	679.660	4.615
MAT + MAP + *P*_cold_	0.997	0.983	680.982	5.937
MAP + *P*_cold_ + *P*_warm_	0.992	0.992	683.237	8.192
MAT + *P*_warm_ + *P*_cold_	0.987	0.987	687.386	12.341
P_cold_ + *P*_warm_	0.988	0.993	689.343	14.298
MAP + *P*_warm_	0.992	0.986	693.554	18.509
MAP + *P*_cold_	0.995	0.987	694.870	19.825
MAT + MAP	0.993	0.962	697.212	22.167
MAT + *P*_cold_	0.971	0.942	712.348	37.303
MAT + *P*_warm_	0.979	0.978	725.403	50.358
MAP	0.925	0.827	759.367	84.323
*P*_cold_	0.936	0.863	761.531	86.486
MAT	0.911	0.905	768.760	93.715
*P*_warm_	0.968	0.963	769.818	94.773

Abbreviations: MAP, mean annual precipitation (mm); MAT, mean annual temperature (°C); *P*_cold_, precipitation of coldest quarter (mm); *P*_warm_, precipitation of warmest quarter (mm).

### 2.3 Statistical Analysis and Mapping

**FIGURE 1:**
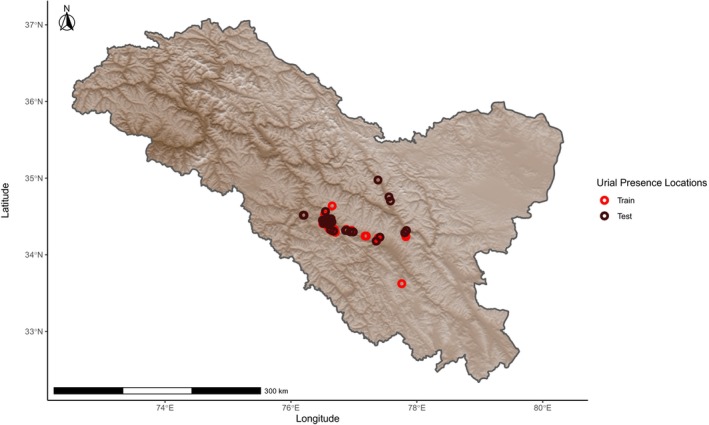
Locations of Ladakh urial occurrence observations after spatial thinning, grouped by random assignment into train and test data subsets used in model building and evaluation, respectively.

**FIGURE 2:**
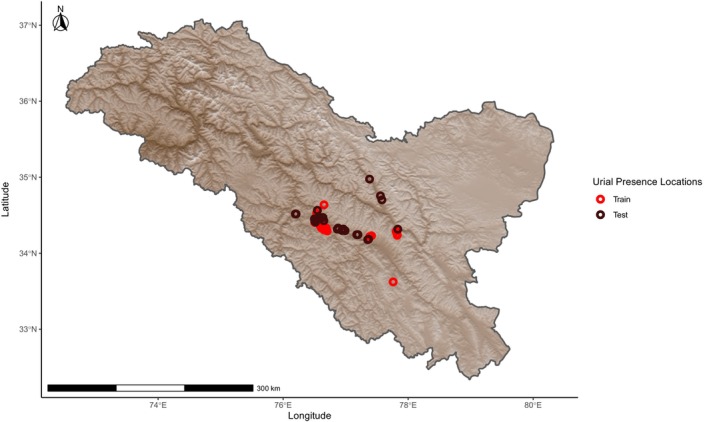
Locations of Ladakh urial occurrence observations after spatial thinning, grouped relative to the median occurrence elevation into train and test data subsets used in model building and evaluation, respectively.

**FIGURE 3:**
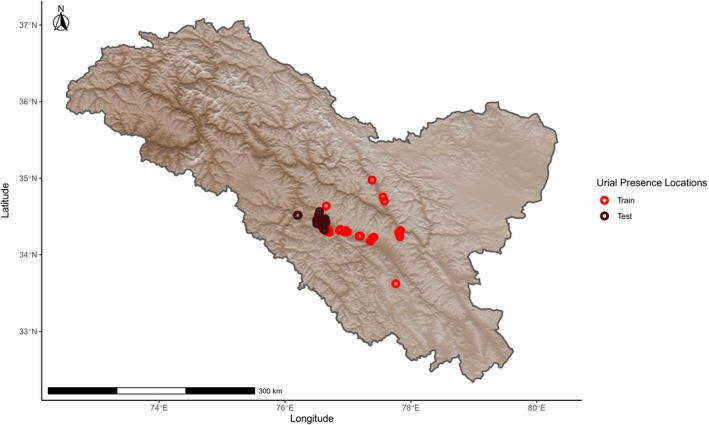
Locations of Ladakh urial occurrence observations after spatial thinning, grouped relative to the median occurrence longitude into train and test data subsets used in model building and evaluation, respectively.

**FIGURE 4:**
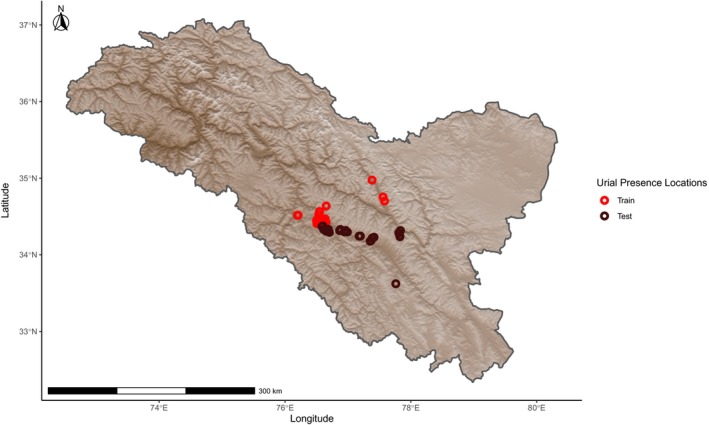
Locations of Ladakh urial occurrence observations after spatial thinning, grouped relative to the median occurance latitude into train and test data subsets used in model building and evaluation, respectively.

Urial distribution was modeled by assigning probabilities of relative suitability to climatic variables at a given location based on presence‐only data using the Maximum Entropy (Maxent) method as part of the dismo package version 1.3‐9 in R version 4.1.1 (Phillips, Dudík, and Schapire 2004; Phillips, Anderson, and Schapire 2006; Elith et al. 2011; Feng, Gebresenbet, and Walker 2017; R Core Team 2021; Hijmans et al. 2022). Prior to constructing models, we ensured that locations of urial presence were at least 1 km from each other to avoid sampling the same discrete cell multiple times with regard to the 30 arc sec (~1 km using the WGS 84 projection at mid‐latitudes) resolution of the model. This was done using the R package spThin version 2.9‐1 (Aiello‐Lammens et al. 2015). Urial environmental suitability models were overlayed with an elevation model created from worldclim elevation data and cropped to within the region of Ladakh sourced from Community Created Maps of India (www.datameet.org). We created environmental suitability models using all combinations of climatic variables relevant in defining cold desert and cold steppe climates, with a focus on combinations that best represented the two forementioned climates. The models were built, and the influence of fine‐scale local variation was evaluated by randomly assigning 50% of the occurrence locations to a subset used to train the models, as mountains are typically characterized by highly heterogeneous environments that generate a wide range of climatic conditions within small geographic areas (Chevalier et al. 2021). A second subset with which the models were tested used the remaining occurrence points (Figure 1). Localized variation from the random sampling was compared with and assessed by training a parallel set of models on a subset of occurrence data that fell above the median elevation of occurrence locations. The points below this value were used for testing (Figure 2). Two more parallel sets of models were also trained and tested on two configurations of spatially independent data subsets to address potential spatial autocorrelation across larger geographic scales. These spatially independent subsets were created by evenly splitting the data used for training around the median occurrence coordinate longitude (Figure 3) or latitude (Figure 4) with the remaining points used to evaluate the models' accuracies, respectively. Within a 400 km buffer of the thinned training occurrence data that covered an area on the same order of magnitude as the Ladakh region, 10,000 background points were randomly sampled. The accuracy of each model was evaluated by calculating the area under the curve (AUC) of its receiver operating characteristic using the respective subset of data for training and testing with the evaluate function of the dismo package. The comparison of test and train AUCs was used to assess whether overfitting had occurred and therefore gauge the model's ability to generalize. Models were ranked based on their ability to effectively describe the underlying pattern of the data while penalizing unnecessary parameters using Akaike information criterion corrected for sample size (AICc) with the full dataset of urial locations using the R package ENMeval version 2.0.4 (Warren and Seifert 2011; Feng, Gebresenbet, and Walker 2017; Kass et al. 2021).

The True Skill Statistic (TSS) calculated with dismo's evaluate function was used to apply a threshold with dismo's threshold function that defined the maximum extent of predicted urial presence for the modeled niche (Allouche, Tsoar, and Kadmon 2006; Qiao et al. 2019; Hijmans et al. 2022). From within the bounds of the TSS threshold, the environmental suitability was further categorized as high, moderate, low, and fringe. This was done by retaining a percentage of the highest values within the TSS threshold, where high outlines the upper 30% of suitable habitat, moderate shows the upper 70% of suitable habitat, and low is the retention of the upper 90% of TSS values. Fringe environment was classified as environments of suitability > 90% of the most suitable habitat. All values outside the TSS threshold were classified as unsuitable habitat. The values used in this categorization are based on the lucky number sieve, which eliminates neighboring natural numbers asymptotically (Gardiner et al. 1956).

## 3 Results

Urial presence was recorded at 164 locations in Ladakh over 65 survey days during the northern autumn, winter, and spring months in the years 2019, 2020, 2022, and 2023. After spatial thinning, we were left with 50 locations with which the models were built. The train and test AUC values were comparable across all train and test sampling configurations with the exception of elevation which saw a larger discrepancy between test and train AUC values (Tables 1, 2, 3, 4). The top model based on AIC_c_ ranking created from a random training data subset used all climatic variables and demonstrated a high level of accuracy with a train AUC of 0.997 (Table 1). The test AUC of the top model was 0.995, and with a similar value to that of the training AUC demonstrated that it could generalize well and is not overfitting the training data (Table 1). The model that used the cold steppe parameters MAT, MAP, and *P*_warm_ ranked 2^nd^ with an AICc of 4.615 above the top model and was highly accurate with a train AUC of 0.997 and a test AUC of 0.997 (Table 1). The model that best represents our cold desert hypothesis using MAT, MAP, and *P*_cold_ parameters also performed well, ranking 3^rd^ with an AICc value that was 5.937 above that of the top‐ranked model as well as showed highly accurate train and test AUCs of 0.997 and 0.983, respectively (Table 1).

Across the spatially thinned locations, the average MAP was 133 mm and was skewed toward lower values (skewness = 1.695) with a median of 126 mm (Table 5). The *P*_cold_ parameter averaged higher and skewed left ($\bar{x}$ = 45 mm, $\tilde{x}$ = 42 mm, skewness = 1.633) relative to the lower averaging and also left skewed *P*_warm_ parameter ($\bar{x}$ = 24 mm, $\tilde{x}$ = 22 mm, skewness = 1.375) over the spatially thinned locations (Table 5). The MAT distribution was parametric (skewness = 0.070) with a mean value of 2.8°C (Table 5). A greater number of locations where urial were observed fell within the highly suitable environment (21%) as classified by the BWk representative model than within this category (10%) of the BSk model (Table 3). The BWk model classified fewer locations as fringe environment (26%) than did the BSk model (35%) (Table 3). However, the BSk model classified fewer sites as unsuitable environment (4%) compared to the BWk model (9%) (Table 3). Generally, The lower‐quality environments saw a broader range of precipitation values that spanned both above and below those of higher‐quality environments as shown by the differences in standard deviations (Table 5).

### 3.1 Köppen‐Geiger Climate Associated Distribution

As categorized by the Köppen‐Geiger climate classification outlined in Peel, Finlayson, and McMahon (2007), the climate in which urial were most frequently observed was cold steppe and comprised 96 (59%) of all presence locations. Cold desert climate followed with urial observed at 51 (31%) of all locations, and lastly, 17 (10%) observations fell within other climate categories. Among the spatially thinned locations, the BSk climate was classified at a greater rate with 36 (72%) of the locations that fell into this category, compared to the 10 (20%) that were classified as BWk, and 4 (8%) that were classified as other climates.

### 3.2 Cold Desert Model Distribution Map

**FIGURE 5:**
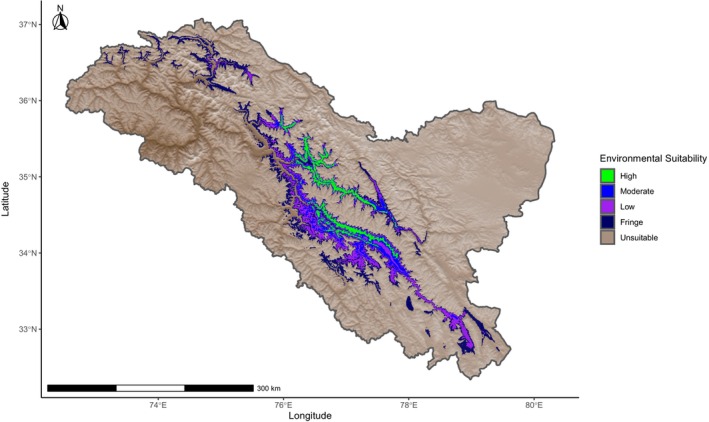
Ladakh urial environmental suitability model within the region of Ladakh created from a random subset of urial occurrence locations and used mean annual temperature, mean annual precipitation, and precipitation of coldest quarter parameters that are representative of cold desert climate (BWk). Environmental suitability is categorized as high, moderate, low, and fringe from a retained percentage of the True Skill Statistic with all values falling outside this threshold classified as unsuitable.

The most suitable potential habitat mapped through the BWk model with randomly assigned points shows the core areas along the Indus and Shayok rivers. It extends toward Siachen Glacier up to Sumur village along the Nubra River. Along the Shayok River, the most suitable environment starts at Thang at the border with Pakistan and ends around the village of Digar, whereas along the Indus, the highly suitable environments starts around the Batalik area in western Ladakh and ends around Kharu in eastern Ladakh. The model also identified areas of high environmental suitability further west in Gilgit‐Baltistan (Figure 5). The most suitable potential habitat in this region is along the Shyok River extending into the Hushe and Saltoro river valleys. These areas, however, were not surveyed during this study.

Along the Indus, less suitable environments (moderate and low) extend eastward to the village of Himya. These potential habitats follow tributary streams and rivers into the interior parts of this watershed. For instance, it extends along the Wanla stream up to Sirsir La pass. Along the Zanskar River, it extends slightly beyond the village of Chilling, and along the Markha stream it extends up to Nimaling.

### 3.3 Cold Steppe Model Distribution Map

**FIGURE 6:**
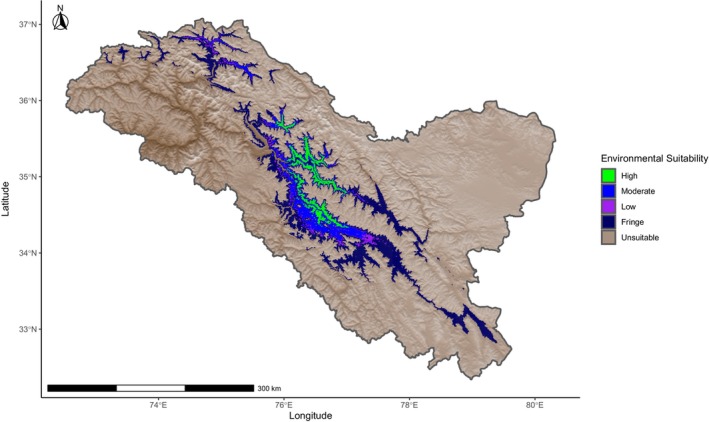
Ladakh urial environmental suitability model within the region of Ladakh created from a random subset of urial occurrence locations and used mean annual temperature, mean annual precipitation, and precipitation of coldest quarter parameters that are representative of cold steppe climate (BSk). Environmental suitability is categorized as high, moderate, low, and fringe from a retained percentage of the True Skill Statistic with all values falling outside this threshold classified as unsuitable.

**FIGURE 7:**
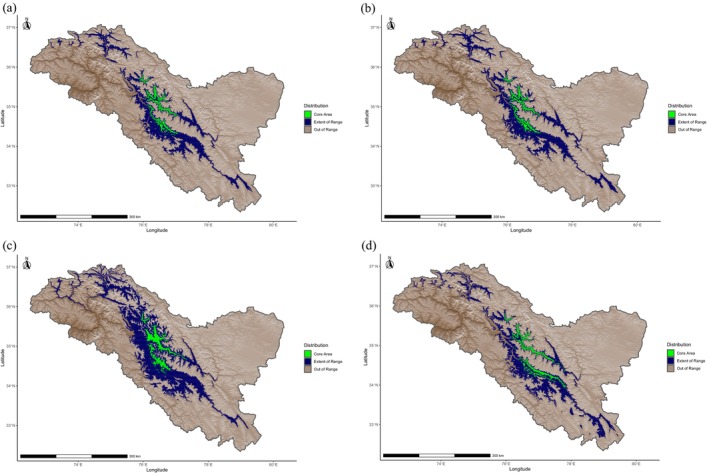
Ladakh urial environmental suitability models within the region of Ladakh created from (a) mean annual temperature, mean annual precipitation, precipitation of coldest quarter and precipitation of warmest quarter parameters; (b) mean annual temperature, mean annual precipitation and precipitation of warmest quarter parameters representative of cold steppe climate (BSk); (c) mean annual precipitation, precipitation of coldest quarter and precipitation of warmest quarter parameters; (d) mean annual temperature, mean annual precipitation and precipitation of coldest quarter parameters representative of cold desert climate (BWk). Distribution is categorized into core area or extent of range, with all areas beyond the extent of range outlined by the True Statistic threshold out of range.

The map resulting from the BSk model using randomly assigned points showed the higher quality potential habitats further northwest in the wider section of the Indus and Shyok valleys and absent in the Nubra Valley. This model did not show the urial's range extending down into the narrow portion of the Indus Valley nor as far west into the Zanskar Range (Figure 6). The top‐ranked model had a comparable core area and range with the BSk model, other than not having fringe environments extend as far north into the Nubra Valley (Figure 7).

## 4 Discussion

In this study, the environmental preferences of the Ladakh urial were examined through a series of models that aligned the animal's occurrence with fine‐scale climate data to test the hypothesis that this species has an affinity for arid conditions. These models have demonstrated that the transitional zone between the cold desert and cold steppe climates of Ladakh contributes considerably to describing the potential habitat and range of urial in this region. The environments suitable for Ladakh urial identified through modeling overlap well with the current distribution of the species established through anecdotal accounts. The study's results show that the selected climatic variables adequately explain the distribution pattern of the Ladakh urial in the Indian Trans‐Himalaya. Winter precipitation in the form of snow seems to influence the distribution of the animal, while high precipitation during summer in the form of rain is indirectly involved in describing its distribution. This is likely because the vegetation growth in much of Ladakh is influenced by glaciers and snowpack, which melt gradually allowing water to seep into the ground and maintaining a constant level of soil moisture through the short growing season. Heavy rain during summer may either support areas with more uniform and continuous steppe grasslands with a higher prevalence of grazers specialized to these environments or wash away the substrates from steeper mountain slopes, thereby leading to the degradation of sufficiently suitable habitat for urial.

From the climatic conditions examined, the climate that best described the urial's niche is that of a cold arid zone more broadly, marked by limited precipitation and temperatures reaching below freezing for much of the year (Peel, Finlayson, and McMahon 2007; Table 5). Although the purely descriptive outline of the urial's distribution from the overlain Köppen‐Geiger climate categories showed that urial are more often found in cold steppe climates, cold desert climates also made up a large portion of the area used by urial, and our modeling suggests that it plays a crucial role in expressing core areas with preferred environmental conditions. The cold desert model accounts for the high proportion of cold precipitation to annual precipitation with *P*_cold_ and MAP parameters, as well as temperature through the MAT parameter. Temporal temperature differences were not compared to MAT as this relationship is not used in defining arid environments. However, deserts typically see large diurnal ranges in temperature, as well as dramatic seasonal temperature variation in cold deserts with implications on the phase of precipitation, and these patterns may warrant further exploration (Ward 1906; Dai and Wang 2017). Elevation was identified as having the largest discrepancy between test and train AUCs of any spatial arrangement tested, with MAT having the most pronounced difference among the tested variables (Table 2). However, these results were expected given the strong inverse relationship between temperature and elevation (Whiteman 2000). Overall, the BWk model was well suited for identifying core areas and classified a greater number of locations at which urial were observed as high quality environments compared to other high‐ranking models (Table 6).

**TABLE 2:** Urial distribution models with accuracy evaluation using the Area Under Curve (AUC) of Train and Test data subsets with positive spatial autocorrelation grouped in relation to elevation, and model ranking using Akaike Information Criterion corrected for sample size (AIC_c_) and the difference from the top model (ΔAIC_c_).

Model	AUC Train	AUC Test	AIC_c_	Δ AIC_c_
MAT + MAP + *P*_cold_	0.999	0.904	662.470	0.000
MAT + MAP + *P*_cold_ + *P*_warm_	0.998	0.913	663.652	1.182
MAT + *P*_warm_ + *P*_cold_	0.997	0.911	668.080	5.609
MAT + MAP + *P*_warm_	1.000	0.929	675.333	12.863
MAT + *P*_cold_	1.000	0.826	683.182	20.712
MAT + MAP	0.988	0.865	685.671	23.201
MAP + *P*_cold_ + *P*_warm_	0.994	0.990	686.552	24.082
*P*_cold_ + *P*_warm_	0.978	0.980	694.439	31.969
MAP + *P*_cold_	0.993	0.967	697.266	34.796
MAT + *P*_warm_	0.984	0.888	700.945	38.475
MAP + *P*_warm_	0.989	0.984	702.338	39.868
MAT	0.959	0.757	737.223	74.753
*P*_cold_	0.936	0.841	752.299	89.829
MAP	0.909	0.802	757.957	95.487
*P*_warm_	0.957	0.975	778.466	115.996

Abbreviations: MAP, mean annual precipitation (mm); MAT, mean annual temperature (°C); *P*_cold_, precipitation of coldest quarter (mm); *P*_warm_, precipitation of warmest quarter (mm).

**TABLE 3:** Urial distribution models with accuracy evaluation using the Area Under Curve (AUC) of Train and Test data subsets with large‐scale positive spatial autocorrelation along an east–west split, and model ranking using Akaike Information Criterion corrected for sample size (AIC_c_) and the difference from the top model (Δ AIC_c_).

Model	AUC Train	AUC Test	AIC_c_	Δ AIC_c_
MAT + MAP + *P*_cold_	0.986	0.981	641.406	0.000
MAT + MAP + *P*_cold_ + *P*_warm_	0.992	0.995	682.057	40.650
MAP + *P*_cold_ + *P*_warm_	0.987	0.989	689.805	48.398
MAT + *P*_warm_ + *P*_cold_	0.979	0.994	693.354	51.948
*P*_cold_ + *P*_warm_	0.982	0.997	698.284	56.878
MAP + *P*_cold_	0.989	0.978	701.659	60.253
MAT + MAP + *P*_warm_	0.994	0.997	705.316	63.909
MAT + *P*_cold_	0.961	0.940	717.059	75.653
MAT + MAP	0.982	0.976	720.273	78.867
MAP + *P*_warm_	0.982	0.995	729.467	88.061
MAT + *P*_warm_	0.973	0.991	734.160	92.754
*P*_cold_	0.923	0.867	765.079	123.673
MAT	0.903	0.914	774.281	132.875
*P*_warm_	0.933	0.979	781.520	140.114
MAP	0.874	0.857	795.663	154.257

Abbreviations: MAP, mean annual precipitation (mm); MAT, mean annual temperature (°C); *P*_cold_, precipitation of coldest quarter (mm); *P*_warm_, precipitation of warmest quarter (mm).

**TABLE 4:** Urial distribution models with accuracy evaluation using the Area Under Curve (AUC) of Train and Test data subsets with large‐scale positive spatial autocorrelation along a north–south split, and model ranking using Akaike Information Criterion corrected for sample size (AIC_c_) and the difference from the top model (Δ AIC_c_).

Model	AUC Train	AUC Test	AIC_c_	Δ AIC_c_
MAT + MAP + *P*_cold_	0.997	0.993	641.065	0.000
*P*_cold_ + *P*_warm_	1.000	0.974	642.873	1.808
MAP + *P*_cold_ + *P*_warm_	1.000	0.974	644.948	3.884
MAT + *P*_warm_ + *P*_cold_	1.000	0.977	652.379	11.314
MAT + MAP + *P*_cold_ + *P*_warm_	1.000	0.977	654.481	13.416
MAP + *P*_warm_	1.000	0.965	665.895	24.830
MAT + MAP + *P*_warm_	0.999	0.973	677.168	36.103
MAT + *P*_warm_	0.996	0.968	690.401	49.336
MAP + *P*_cold_	0.997	0.985	704.962	63.897
MAT + *P*_cold_	0.956	0.940	724.837	83.772
*P*_warm_	0.960	0.908	725.921	84.856
MAT + MAP	0.974	0.967	726.205	85.140
MAT	0.937	0.891	758.155	117.091
*P*_cold_	0.900	0.919	791.835	150.770
MAP	0.876	0.894	809.434	168.369

Abbreviations: MAP, mean annual precipitation (mm); MAT, mean annual temperature (°C); *P*_cold_, precipitation of coldest quarter (mm); *P*_warm_, precipitation of warmest quarter (mm).

**TABLE 5:** Summary of climatic variables at urial locations after spatial thinning (*n* = 50).

Climatic Variable	Min	Max	Mean	Median	SD
MAP	60	296	133	126	45
MAT	−2.7	7.1	2.8	2.9	2.7
*P*_cold_	17	121	45	42	22
*P*_warm_	16	45	24	21	7
*P*_winter_	27	204	75	70	38
*P*_summer_	32	92	57	56	11

Abbreviations: MAP, mean annual precipitation (mm); MAT, mean annual temperature (°C); *P*_cold_, precipitation of coldest quarter (mm); *P*_warm_, precipitation of warmest quarter (mm); *P*_winter_, precipitation during the 6 month period of ONDJFM (mm); *P*_summer_, precipitation during the 6 month period of AMJJAS (mm).

**TABLE 6:** Summary of climatic variables at all urial occurrence locations (*n* = 164) within modeled environmental suitability categories of high, moderate, low, and fringe using parameters representative of cold desert (BWk) and cold steppe (BSk) climates.

Climatic Variable	Mapped BWk			Mapped BSk		
Environmental Suitability	Mean	Median	SD	Mean	Median	SD
**High**		(*n* = 35)			(*n* = 16)	
MAP	134	132	15	135	133	14
MAT	2.1	1.5	1.2	2.5	2.5	1.1
*P*_cold_	46	43	8	48	46	8
*P*_warm_	21	20	2	20	20	1
**Moderate**		(*n* = 28)			(*n* = 59)	
MAP	127	121	23	128	123	21
MAT	1.5	0.5	1.7	1.8	1.1	2.3
*P*_cold_	42	40	12	43	41	12
*P*_warm_	22	22	2	22	22	2
**Low**		(*n* = 44)			(*n* = 25)	
MAP	125	116	23	114	112	10
MAT	4.5	5.2	2.9	4.6	5.1	1.7
*P*_cold_	41	35	15	34	33	7
*P*_warm_	26	25	6	30	30	4
**Fringe**		(*n* = 43)			(*n* = 58)	
MAP	95	93	42	117	96	61
MAT	1.8	1.4	3.1	2.5	3.1	3.4
*P*_cold_	24	21	19	35	21	30
*P*_warm_	29	27	9	29	27	10
**Unsuitable**		(*n* = 14)			(*n* = 6)	
MAP	136	118	93	60	60	0
MAT	2.4	2.5	2.7	2.5	2.5	0
*P*_cold_	43	23	43	17	17	0
*P*_warm_	30	22	11	21	21	0

Abbreviations: MAP, mean annual precipitation (mm); MAT, mean annual temperature (°C); *P*_cold_, precipitation of coldest quarter (mm); *P*_warm_, precipitation of warmest quarter (mm).

The limited water resources available to vegetation in desert climates lead to a less productive landscape. A plant's access to water may be further limited by the cold‐dominated precipitation typical of cold desert ecosystems where it cannot be used or stored by the plant outside of the short growing season (Baudena and Provenzale 2008). Water scarcity generally results in desert landscapes supporting sparsely distributed vegetation that typically has relatively short and short‐lived aboveground structures that aid these plants in retaining water (Tariq et al. 2024). Urial likely outperform other ungulates in this resource‐scarce environment through means that may include targeting lower‐quality vegetation, traveling further between forage, or a combination of these strategies. This approach of opting for lower‐quality food may also contribute to the urial's proclivity to use both flat and vertical terrain as they search for sufficient nutrient and energy resources.

**FIGURE 8:**
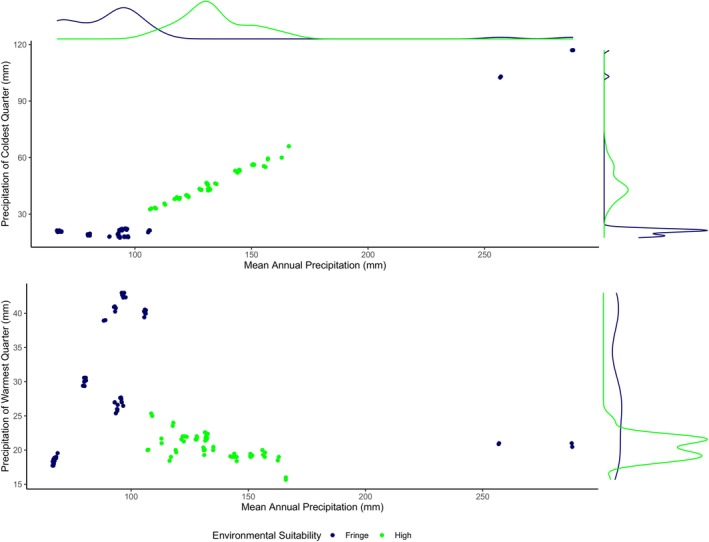
Comparison of the influences that cold precipitation and warm precipitation patterns have on modeled environmental suitability for Ladakh urial. The model in this comparison used mean annual temperature, mean annual precipitation, and precipitation of coldest quarter parameters that are representative of cold desert climate (BWk).

**FIGURE 9:**
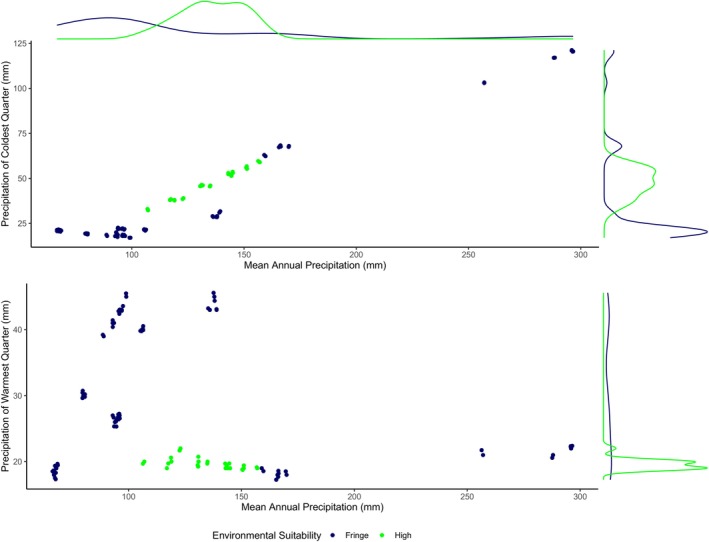
Comparison of the influences that cold precipitation and warm precipitation patterns have on modeled environmental suitability for Ladakh urial. The model in this comparison used mean annual temperature, mean annual precipitation, and precipitation of warmest quarter parameters that are representative of cold steppe climate (BSk).

In addition to strictly considering “desert” variables, the *P*_warm_ parameter was also examined as we believed that it may have aided in isolating the cold desert cold‐dominated precipitation pattern from that of the warm‐dominated precipitation of high‐altitude steppe (Miehe et al. 2011; Lone et al. 2019). In doing so we hoped to further highlight the difference in the potential of primary productivity. This seems to have been the case as this addition resulted in an overall higher ranking of models than did the temperature and *P*_cold_ parameters (Table 1). The cold‐dominated precipitation more typical of the cold desert climates implies a high cold precipitation to annual precipitation ratio and is addressed by the *P*_cold_ parameter (Figure 8). However, this pattern also implies the inverse of a low warm precipitation to annual precipitation ratio. This inverse ratio is not identified by the *P*_cold_ parameter but is captured well by the *P*_warm_ parameter. As the higher quality environments run up against the floor of possible warm precipitation values, the *P*_warm_ parameter very effectively distinguishes these areas from those with greater amounts of warm precipitation (Figure 9). Under steppe climatic conditions, water resources are more available to vegetation during the growing season when it is most needed, readily taken up, and retained (Churkina and Running 1998; Baudena and Provenzale 2008). As a result, steppe habitat produces significantly more biomass that is available to be grazed. Although urial may do well in this steppe habitat, they are typically not found deep within these areas. In this study, urial were observed only on the periphery of the steppe habitat and in close proximity to desert habitat. This *P*_warm_ parameter may serve as a proxy for the edge of the boundary where competition with other herbivores may occur which is a likely reason for the urial's exclusion from these areas. If competition is the reason for the urial's exclusion from core steppe habitat then in the context of the model, the *P*_warm_ parameter could be conceptualized as the realized niche. The BSk model has demonstrated the ability to effectively exclude many of the locations of a more typical BSk climate with overall greater summer and annual precipitation from possible urial habitat and is better able to define the extent of the urial's range than the BWk model (Table 6).

Competition with other ungulates in environments deemed to be lower in their suitability for urial could lead to the exclusion of urial from these areas. Such competition may occur between urial and argali in the upper Indus River valley of southeastern Ladakh where the transition from desert scrub to steppe pastures may be more favorable to argali (Fox, Nurbu, and Chundawat 1991). Likewise, in the Zanskar Range more productive, high alpine pastures may be favorable to blue sheep that drive out the urial through competition (Fox, Nurbu, and Chundawat 1991). In both the previous cases, these areas were categorized as fringe environments where in reality urial may be outcompeted rather than maladapted. In addition to competing with wild ungulates, urial likely face competition from grazing livestock (Raghavan, Bhatnagar, and Qureshi 2003). Unlike wild ungulates that act autonomously, the grazing of domestic animals is governed by human decisions and not by environmental factors alone. Although urial may have been highly restricted in Ladakh by interactions with other wild grazers, they seem nevertheless to be well adapted to the harsh desert environment on the periphery of grazable lands. However, this adaptation of urial also highlights the importance of implementing sustainable grazing and other consumptive land‐use practices in maintaining suitable habitat for urial even in environments with low productivity.

The narrow distribution of urial modeled in the Shyok Valley in contrast to the gradual progression toward lower quality environments southward into the Zanskar Range in all higher ranking models may be explained by the differences in precipitation gradients of local ranges. In the north where the valleys are sheltered by the sharp precipitation gradients of the Karakoram and Ladakh Ranges, the arid climate is limited to the valley, but the rain shadow is drawn out over the leeward side of the Zanskar Range, and this area experiences a much more gradual precipitation gradient (Bhutiyani 1999; Dahri et al. 2016; Jonell et al. 2017; Soheb et al. 2020). These more expansive areas of moderate to fringe environmental suitability (model dependent) to the south of the core Indus area may see the occupation or the disappearance of urial as their population in Ladakh expands or contracts, and these areas may serve as key sites to observe such trends.

This study has outlined the important role cold desert and cold steppe climates play in describing the distribution of the Ladakh urial. It has also highlighted the need for further study of the interactions within this arid ecosystem, both biotic and abiotic, to gain a fuller picture of the niche that urial occupy to properly address potential threats facing both this and other species as well as the arid environments in which they reside.

## Author Contributions

**Jeremy Roy Lambe:** conceptualization (equal), data curation (lead), formal analysis (lead), investigation (supporting), methodology (lead), software (lead), validation (lead), visualization (lead), writing – original draft (lead), writing – review and editing (equal). **Mohd Raza:** investigation (lead), methodology (supporting), writing – review and editing (equal). **Tsewang Namgail:** conceptualization (equal), writing – review and editing (equal).

## Ethics Statement

Urial occurrence data was collected through visual observation and was therefore non‐invasive. Surveys were conducted on public land or with permission from landowners on private land.

## Conflicts of Interest

The authors declare no conflicts of interest.

## Data Availability

The data and code used in the analysis are available at DOI: https://doi.org/10.5061/dryad.cjsxksnfp.
